# Laboratory Test of Industrial Waste Mud Treated by the Flocculation-Vacuum-Curing Integrated Method: Deep Dehydration and Preparation of Geopolymer Fluid Solidified Soil

**DOI:** 10.3390/ma18132961

**Published:** 2025-06-23

**Authors:** Jing Ye, Jingwei Zhang, Peng Zhang, Jia Li, Shanlin Yi

**Affiliations:** 1 School of Water Conservancy and Transportation, Zhengzhou University, Zhengzhou 450001, China; yejing@gs.zzu.edu.cn (J.Y.); zhangpeng@zzu.edu.cn (P.Z.); lijia@zzu.edu.cn (J.L.); 202022222014437@gs.zzu.edu.cn (S.Y.); 2 School of Civil Engineering, Zhengzhou University, Zhengzhou 450001, China

**Keywords:** waste mud, resource reutilization, vacuum filtration combined with electroosmotic dehydration, deep dehydration, geopolymer fluid solidified soil, long-term performance

## Abstract

Resource reutilization of industrial waste mud has encountered challenges due to its high water content, enhanced fluidity, and inherent difficulty in segregating mud and water phases. The author first screened out efficient flocculants through flocculation dehydration tests and then adopted the technology of vacuum filtration combined with electroosmosis dehydration to conduct deep dehydration of waste mud. Among them, the independently designed vacuum filtration electroosmosis system effectively solves the problems of easy clogging and bending of the traditional system. On this basis, geopolymer fluid solidified soil was prepared using dehydrated mud, furnace slag, and fly ash as raw materials, and the influencing factors of its long-term service performance were studied. It was confirmed that the efficient treatment capacity of the combined dehydration technology for industrial waste mud, and the geopolymer fluid solidified soil prepared from dehydrated mud has engineering application potential. This research provides a reference for the resource utilization of industrial waste mud.

## 1. Introduction

With the current development of society, the exponential growth of infrastructure has made the treatment of construction waste an urgent issue that needs to be addressed. Industrial waste mud with high moisture content accounts for a large proportion of this waste. If not properly treated, it will pose a serious threat to the ecosystem [1,2,3,4]. Waste mud is usually treated through external transportation and dumping, chemical flocculation, and physical and mechanical solid–liquid separation, etc., which have problems such as poor environmental protection, poor economy, and low resource utilization rate [5,6]. Therefore, developing innovative technologies to achieve completely zero-pollution utilization of waste mud remains an open research challenge.

Considering the poor environmental protection and significant hazards in the external transportation and dumping treatment, chemical flocculation, and physical-mechanical separation are currently the main methods for treating waste mud. Chemical flocculation achieves the purpose of dehydration by introducing flocculants to destabilize the stability of the mud. Li et al. [7] conducted a flocculant dehydration test on waste mud with high viscosity particle content. The results showed that cationic polyacrylamide (CPAM) and anionic polyacrylamide (APAM) exhibit superior flocculation performance. He et al. [8] investigated the effects of polyacrylamide (PAM) with different charge types and charge densities on the dehydration efficiency of waste mud. The results showed that cationic polyacrylamide (CPAM) with a charge density of 15 yielded the optimal dehydration effect. Gao et al. [9] studied the dehydration effects of different flocculants on waste mud, reporting a descending order of effectiveness: APAM > NPAM > CPAM > PAC. Physical-mechanical separation is the process of separating soil particles from water molecules through techniques such as suction filtration and centrifugation. In recent years, extensive research has been conducted to enhance the efficiency of physical-mechanical dehydration, focusing on the spatial configuration of mechanical devices and combined dewatering methods. Zhang et al. [10] compared the effects of different spatial arrangements of drainage belts on soft soil dehydration, demonstrating that transverse placement effectively shortens the seepage path and improves dehydration efficiency. Liu et al. [11] investigated the combined application of vacuum-electroosmosis filtration to dehydrate soft soil foundations, showing that this method significantly enhances soil shear strength and bearing capacity. Wang et al. [12] explored the enhancing effect of electroosmosis on the dehydration effect of vacuum filtration clay. The results showed that the method of vacuum combined with electroosmosis dehydration could effectively reduce power consumption and improve the effect. Overall, chemical flocculation and physical-mechanical separation can effectively reduce the moisture content of waste mud. However, a large number of international technical standards stipulate that when waste mud is used as building materials, the moisture content should be less than 25% [13,14]. Therefore, it is necessary to conduct relevant research on the deep dehydration of waste mud.

The American Concrete Institute (ACI) defines fluid-solidified soil as a controllable, low-strength material, mainly used for engineering backfilling. A large amount of solid waste from industries such as power generation and smelting provides an opportunity for concrete applications to serve as a geopolymer cementitious agent [15,16,17,18,19]. After deep dewatering, the waste mud can be mixed with curing agents and other additives to prepare fluid, solidified soil. This can effectively solve the problem of waste mud accumulation and achieve the reuse of resources. Research scholars have carried out a large number of studies on the preparation methods and application performance of fluid-solidified soil. Zhu et al. [20] prepared fluid-solidified soil using construction waste soil as raw material and studied its long-term service performance. The results showed that the fluidity of the solidified soil increased with the increase in the water-solid ratio, and its strength increased with the increase in the ash-sand ratio. It is worth noting that there are certain differences between the laboratory tests and the actual on-site construction effect. Compared with the laboratory mixtures, the fluidity of field mixtures decreased by approximately 15%. Kong et al. [15] used red mud instead of cement to prepare fluid-solidified soil. The research results showed that the increase in red mud could reduce the fluidity and bleeding rate of the mixture. Yeong et al. [21] found that the addition of fly ash can improve the workability of fluid-solidified soil, thereby prolonging the setting time. However, the current research on fluid-solidified soil mainly focuses on the study of curing materials and their mechanical properties, lacking research on the curing mechanism and long-term stability of green curing agents for fluid-solidified soil.

Aiming at the problem mentioned above, the author studied the dehydration and consolidation of industrial waste mud and evaluated the performance of geopolymer fluid-solidified soil prepared from dehydrated mud. This research provides valuable insights for achieving sustainable and zero-pollution utilization of industrial waste mud.

## 2. Materials and Schemes

### 2.1. Test Materials

#### 2.1.1. Industrial Waste Mud

The waste mud utilized in this experiment was sourced from a construction project located in Zhengzhou, Henan Province, China. The physical parameters include a relative density of 1.18 g/cm^3^, a water content of 245%, and a colloid content of 70%.

#### 2.1.2. Flocculants

To comprehensively investigate the dehydration effects of different types of flocculants on waste mud, preliminary tests were conducted to compare the performance of various flocculants. Based on the results, four flocculants with superior dehydration capabilities were selected for further study. The organic flocculants included anionic polyacrylamide (APAM) and nonionic polyacrylamide (NPAM), while the inorganic flocculants comprised polymerized aluminum chloride (PAC) and polymerized ferric sulfate (PFS).

#### 2.1.3. Curing Agents and Modifiers

As the curing agents, S95-grade granulated blast furnace slag (BFS) and Class F fly ash were utilized, while the modifier adopted the polycarboxylic acid system JP-01.

### 2.2. Test Schemes

#### 2.2.1. Test of Waste Mud Deep Dehydration

① The test of flocculant dehydration was conducted on the waste mud with the type and dosage of flocculants as variables. Based on the preliminary test result and relevant market demand, the mud volume was fixed at 500 mL. The organic flocculants (APAM/NPAM) were increased from 0.15 g/L to 0.30 g/L with an increment of 0.05 g/L, and the inorganic flocculants (PAC/PFS) were increased from 0.50 g/L to 2.00 g/L with an increment of 0.50 g/L.

**Figure 1:**
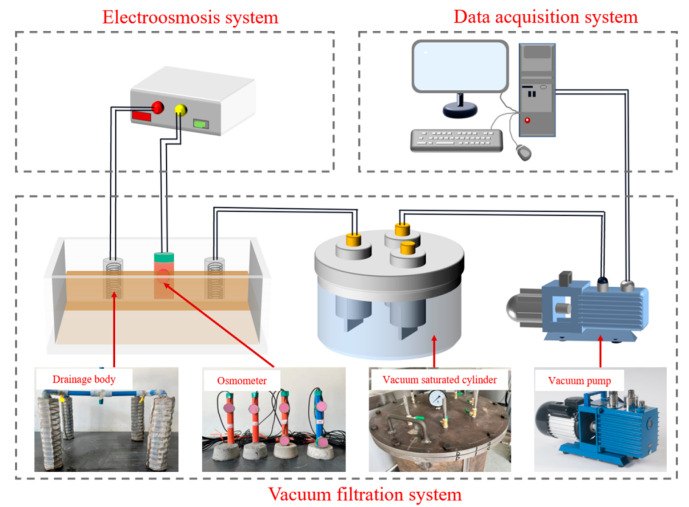
The experimental model device for the waste mud deep dehydration test.

② To achieve deep dehydration of waste mud, the author used a self-designed experimental device (as shown in Figure 1) to conduct the test of vacuum filtration combined with electroosmosis dehydration on the flocculated mud. The device mainly includes a vacuum filtration system, an electroosmosis system, and a data acquisition system, and it can simulate the dehydration effect of mud under different vacuum filtration and electroosmosis by controlling the DC power supply.

Three different schemes were adopted to study the dehydration effect of the technology of vacuum filtration combined with electroosmosis. Among all the schemes, the density of the drainage water body and the drainage path remain consistent. The differences among the three test conditions are specifically reflected in the electroosmosis mode (as shown in Table 1).

**Figure 2:**
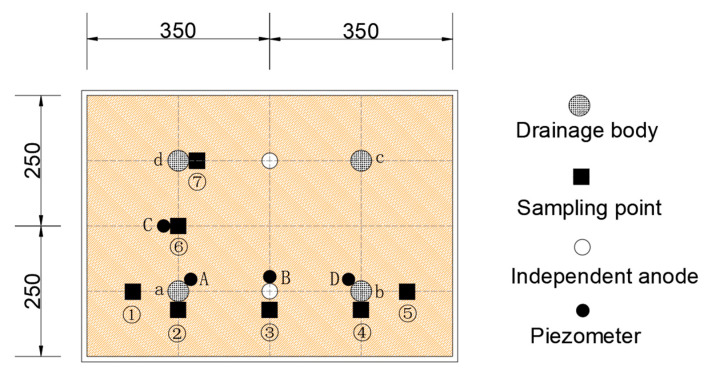
The layout diagram of component in the waste mud deep dehydration test (Unit: mm).

The specific test layout is shown in Figure 2. In the case of ZD1 and ZD2, the drainage bodies a, b, c, and d are the cathodes, and A, B, and C are the piezometers. The vertical distance of the lower pore pressure gauge from the bottom measures 50 mm, while the upper pore pressure gauge is situated 150 mm from the bottom. In addition, the independent anode of the ZD2 group test is located at the midpoint of the two horizontal rows of water. Under ZD3, the left drainage bodies a and d are the cathode, and the right drainage bodies b and c are the compound anode. A, B, and D are piezometers, and the height of the vertical pore pressure gauge is aligned with the heights of the ZD1 and ZD2 groups.

After the test is completed, the consolidated sediment is extracted from the predefined locations (①–⑦). Subsequently, the obtained sediments were divided into upper, middle, and lower layers to facilitate subsequent experimental analysis.

#### 2.2.2. Test of Geopolymer Fluid Solidified Soil Long-Term Performance

**Figure 3:**
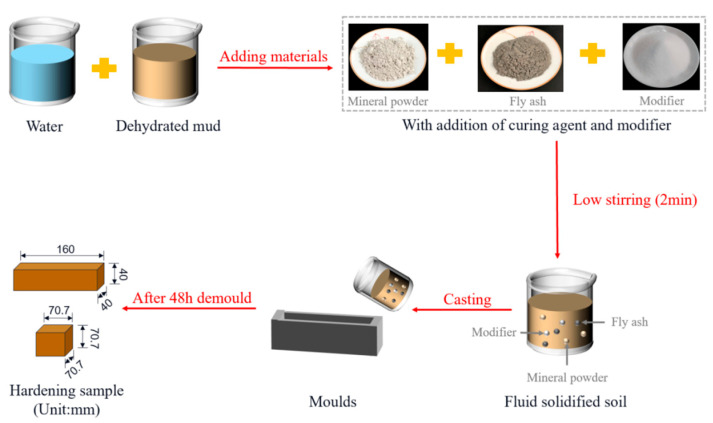
The test process of geopolymer fluid-solidified soil.

In order to achieve the resource utilization of waste mud, the author employed dewatered mud as a cement substitute, combining it with granulated blast furnace slag and fly ash to prepare geopolymer fluid-solidified soil. The overall preparation process of geopolymer fluid-solidified soil is shown in Figure 3.

The author investigates the influence of various factors on the fluidity, unconfined compressive strength, and self-shrinkage performance of geopolymer fluid-solidified soil through gradient experiments. Firstly, the influence laws of the dosage of curing agent and modifier on the self-shrinkage rate of fluid-solidified soil was studied. Then, the influence laws of the dosage of sodium hydroxide, the proportion of furnace slag, and fly ash on the compressive strength and fluidity of fluid-solidified soil were studied. The specific test scheme is shown in Table 2 and Table 3.

## 3. Results and Discussions

### 3.1. Analysis of the Test of Flocculant Dehydration

**Figure 4:**
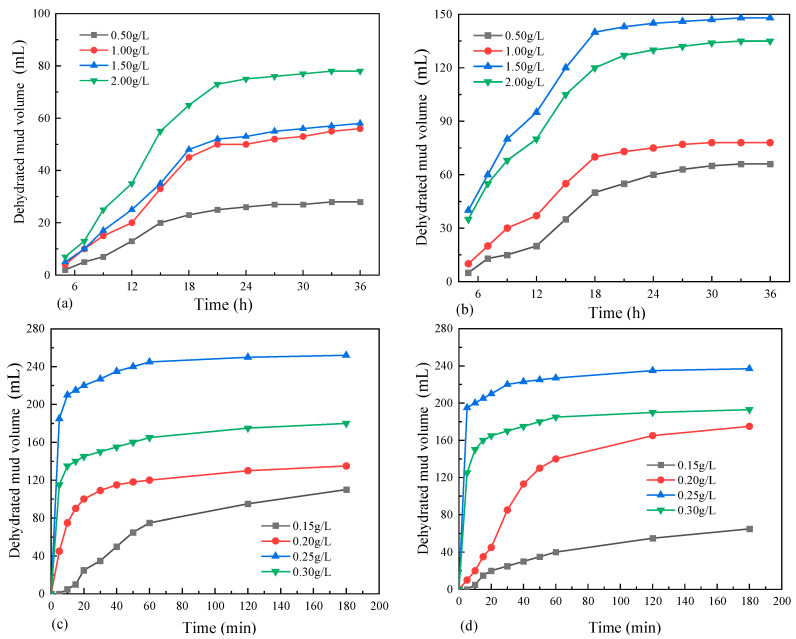
The curve of dehydrated mud volume vs. time: (**a**) PAC; (**b**) PFS; (**c**) APAM; (**d**) NPAM.

Figure 4 shows the relationship curve between the dehydrated mud volume and time under the action of different flocculants. According to Figure 4a,b, the dewatering process of waste mud under the action of inorganic flocculants can be divided into three stages. The first 5 h of the overall dewatering process is a stagnation phase where the dehydration is poor. During this phase, the inorganic flocculant undergoes an initial chemical reaction with the alkaline waste mud yet fails to destabilize the colloidal structure within the mud system. Consequently, minimal water separation was observed. Furthermore, 5–18 h is the mud-water separation stage, when the ionization flocculant ionizes to produce metal cations. These metal cations neutralize the negative charges in the waste mud, thereby weakening the adsorption effect of soil particles on water and accelerating the separation of mud and water. The dehydrated mud volume at this stage accounts for more than 85% of the total. Thereafter, the dehydrated mud volume was basically stabilized.

It can be seen from Figure 4c,d that the dewatering process of waste mud under the action of organic flocculants can also be divided into three stages. The rapid dewatering of the waste mud occurs in the first 10 min of the entire process. After adding the flocculant to the mud, the polymer chains rapidly stretch in the water under the repulsive force of the -COO- group, and then the mud and water are rapidly separated. The dehydration effect at this stage is closely related to the concentration of the flocculant. The period from 10 to 60 min is the flocculation and compaction stage of the mud, during which the rate of mud–water separation gradually slows down. The main reason for this phenomenon is that organic flocculants contain a large number of polar groups with strong adsorption capacity. These groups cause the fine clay particles to coagulate into a network structure through adsorption. These flocculants separate from water molecules under the effect of their own weight, thereby achieving the purpose of dehydration. The dehydrated mud volume remained stable after 60 min.

By comparing the dehydrated mud volume under the action of different flocculants, it can be clearly found that the flocculation treatment of waste mud with organic flocculant APAM has an effective dewatering effect. With the increase in the concentration of the flocculant, the separation rate of mud–water improves. However, excessive flocculants will weaken the adsorption force between molecules, resulting in a decrease in dehydration efficiency. When the dosage of the organic flocculant APAM is 0.25 g/L, the moisture content of the waste mud is reduced from the original 245% to 90.6%, and at this time, the dewatering effect of the mud is the best.

### 3.2. Analysis of the Test of Vacuum Filtration Combined with Electroosmotic Dehydration

#### 3.2.1. Dehydrated Mud Volume

**Figure 5:**
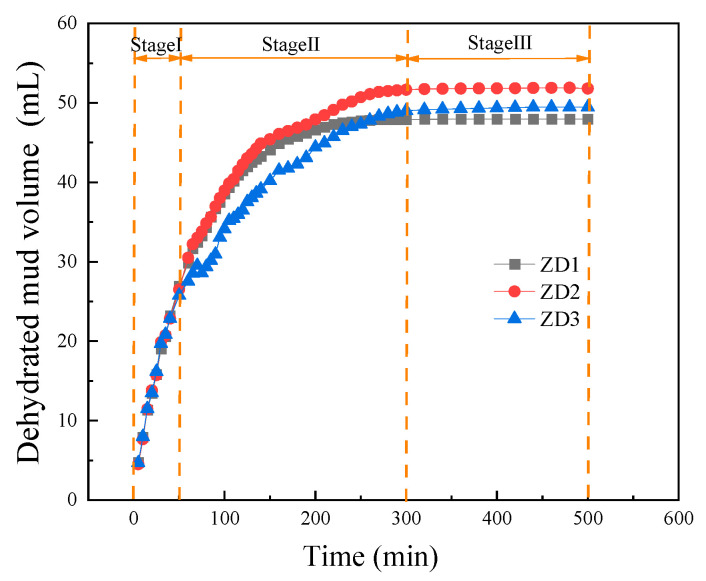
The curve of dehydrated mud volume vs. time.

It can be seen from Figure 5 that the flocculated mud has undergone three stages under different vacuum electroosmosis treatment methods: rapid dewatering, gradual consolidation, and consolidation stabilization. Stage I is the rapid dewatering process of the mud, which occurs within the first 60 to 70 min of vacuum filtration. The dehydrated mud volume in this stage accounts for 40% of the total process. Stage II is the gradual consolidation of the mud. The dehydrated mud volume in this stage accounts for 55% of the total process. The consolidation time of flocculated mud varies under different vacuum electroosmotic treatment methods. The consolidation time of the ZD1 experimental group was approximately 130 min, while that of both the ZD2 experimental group and the ZD3 experimental group was 230 min. Stage III is the consolidation stabilization process of the mud. The dehydrated mud volume under the three conditions was stabilized at 47.96 L, 51.80 L, and 49.45 L, respectively. This indicates that with the extension of consolidation time, the dehydration degree of the flocculated mud shows a downward trend and eventually reaches a stable state.

Compared with the test of vacuum filtration dehydration in group ZD1, the dehydrated mud volume in the experimental groups ZD2 and ZD3 increased by 8% and 3.2%, respectively. This is because the flocculated mud belongs to the soil–water–electrolyte system after being energized, and the water in the mud forms hydraulic seepage with the current. Under the effect of hydraulic seepage, the water molecules accumulated near the cathode in the mud are discharged, resulting in a decrease in the water content of the mud. On the other hand, under the action of electric current, soil particles undergo electrophoresis, reducing the binding ability of soil particles to water molecules [22,23,24].

It is worth noting that the dehydrated mud volume in the ZD2 experimental group was higher than that in the ZD3 experimental group. This phenomenon is caused by the different directions of the electrodialysis water flow and the suction filtration water flow. Under the ZD2 test conditions, the electrodialysis water flow and the suction filtration water flow are in the same direction. The synergistic effect of the two enables the pore water flow to be discharged from the water body. On the contrary, under the ZD3 test conditions, the pore water flow formed by electroosmosis and suction filtration flows in opposite directions at the anode discharge water body, which, to a certain extent, hinders the flow of pore water.

#### 3.2.2. Pore Water Pressure

**Figure 6:**
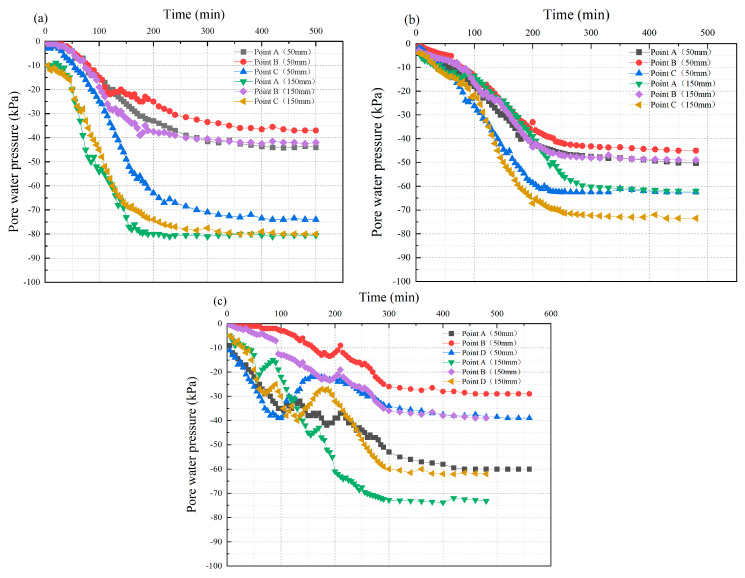
The curve of dehydrated mud pore water pressure vs. time: (**a**) ZD1; (**b**) ZD2; (**c**) ZD3.

Figure 6 presents the relationship curve between the pore water pressure of the flocculated mud and time under different test conditions. The variation of pore water pressure in flocculated mud can be divided into three stages: gradual dissipation, rapid dissipation, and stable dissipation. The pore water pressure dissipation value of the flocculated mud in the early stage is approximately 15% of the entire process, and the duration is about 70 min. As the test time increased, the pore water pressure of the flocculated mud dissipated rapidly, accounting for approximately 80% of the entire process. Subsequently, the pore water pressure of the flocculated mud remained stable.

The observed phenomenon can be attributed to the following reasons: The flocculated mud exhibits a distinct “two-phase flow” structure due to its high free water content. During the early stage of the experiment, the total stress of the soil particles was mainly supported by the pore water pressure, and the dissipation of the pore water pressure was due to the reduction in free water in the structure. As the test time increased, the free water in the flocculated mud was rapidly discharged, and the soil particles came into contact with each other. The increase in effective soil stress leads to a rapid decrease in the pore water pressure of the mud. When the flocculated mud reaches a certain density, the soil permeability coefficient is relatively small, and the pore water pressure remains stable.

It is notable that the test curve of group ZD3 showed an unstable phenomenon at 200 min but returned to normal in a short period of time. The reason for this phenomenon is that under the combined effect of electroosmosis, the soil particles will be adsorbed near the anode of the discharge water body. These soil particles will clog the filter membrane of the drainage water body, causing a temporary increase in the pore water pressure of the flocculated mud. However, as the consolidation reaction proceeds, new fixed pore drainage channels will form at the filter membrane, further reducing the pore water pressure of the flocculated mud.

By comparing the variation curves of pore water pressure of the flocculated mud under the different test conditions, it can be found that the dissipation rate of flocculated mud pore water pressure gradually decreases with the increase in the radial distance and vertical depth of the discharge water body. This phenomenon may be attributed to the influence that the drainage effect is constrained by the vacuum transfer boundary conditions. With the increase in the vertical depth of the drainage body, the density of the flocculated mud further decreases. The decline in the permeability of the mud hinders the effective discharge of pore water.

#### 3.2.3. Water Content of Sediment

**Figure 7:**
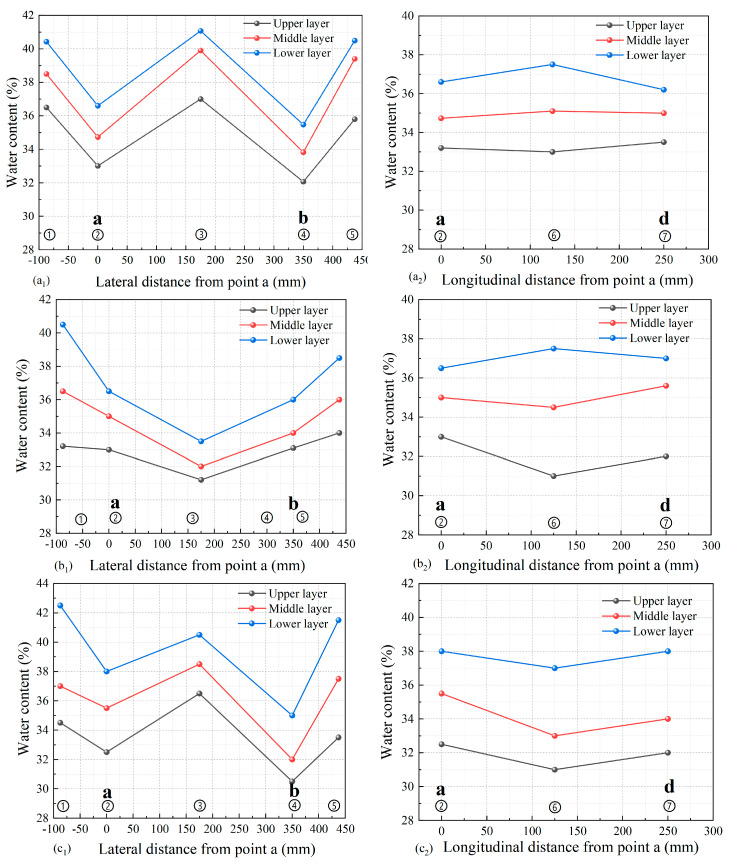
The distribution curve of flocculated mud sediment water content: (**a1**,**a2**) ZD1, (**b1**,**b2**) ZD2, (**c1**,**c2**) ZD3. ①–⑦ are the sampling positions set according to the test protocol. (a,b,d) correspond respectively to the distribution positions of the drainage bodies.

As shown in Figure 7, the moisture content of the flocculated mud after different vacuum filtration combined with electroosmosis dewatering is maintained at 30–40%. Under the different test conditions, the water content distribution characteristics of the flocculated mud at different positions show significant differences. For example, with the increase in the lateral distance, the water content of the mud in the ZD1 and ZD3 experimental groups showed a “W” pattern, while that in the ZD2 experimental group showed a “C” pattern. However, with the increase in the longitudinal distance, the water content of different experimental groups was uniformly distributed.

The reasons for the different water content distribution characteristics of the flocculated mud under the different test conditions are as follows: the water content distribution of the flocculated mud in the transverse direction is mainly affected by the electroosmotic anode. In the ZD2 experimental group, the water content of the flocculated mud at position ③ was the lowest (approximately 31.5–34%). This is because this position is close to the anode. The electroosmotic effect promotes the migration of pore water in the mud towards the vicinity of the discharge water body, thereby significantly reducing the water content of the mud. Considering that in the ZD2 experimental group, the anode was located in the middle of the two drainage bodies, under the combined action of negative pressure water flow and electroosmotic water flow, the water content distribution of the mud presented a “C” shape.

The water content distribution of the flocculated mud in the ZD1 and ZD3 experimental groups was approximately the same, but the water content of the mud at position ④ in ZD3 was lower than that in ZD1. This is because soil particles gradually migrate towards the anode under the effect of electrophoresis, while water molecules migrate towards the cathode. The opposite movement of the two leads to a decrease in the water content of the mud.

### 3.3. Analysis of the Test of Geopolymer Fluid Solidified Soil Long-Term Performance

#### 3.3.1. The Impact Factors of Fluid Solidified Soil’s Fluidity

**Figure 8:**
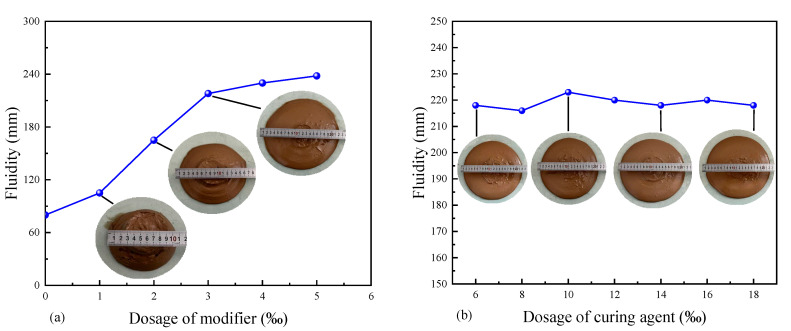
The curve of solidified soil fluidity vs. additives dosage: (**a**) modifier; (**b**) curing agent.

The curve of fluid solidified soil fluidity varies vs. content of additives is shown in Figure 8. The fluidity test of fluid-solidified soil was conducted by referring to the standard test method of CLSM in the United States. The standard stipulates that a cylindrical mold with a diameter of 75 mm and a height of 150 mm should be placed on a smooth and non-absorbent glass plate. The fluid-solidified soil should be poured into the cylindrical cylinder to a height of 150 mm. The cylindrical cylinder should be lifted at a constant speed. When the fluid-solidified soil freely expands to a stop, the horizontal and vertical diameters of the circular expansion surface should be measured. The average is the fluidity of the fluid-solidified soil.

(1) Modifier

The relationship curve of fluid-solidified soil fluidity varies with the content of modifier JP-01, which is shown in Figure 8a. It can be clearly observed that with the increase in the modifier content, the growth rate of the fluidity of the solidified soil shows a changing trend of first increasing and then decreasing. When the dosage of the modifier increased from 0‰ to 3‰, the fluidity of the solidified soil showed a linear increase, increasing by 181% compared with the original state. When the dosage of the modifier exceeds 3‰, the increment of the fluidity of the solidified soil decreases significantly. This indicates that a modifier dosage of 3‰ is a significant change point in the fluidity of solidified soil.

The addition of the modifier significantly enhanced the fluidity of the fluid-solidified soil. This is because the fluid-solidified soil hydrolyzes to produce Ga^2+^ and Na^+^, causing the soil particles to carry positive charges on their surfaces. There are a large number of anions in the modifiers, which can effectively adsorb the solidified soil through two mechanisms: surface adsorption [25] and chemical reaction intercalation [26]. The mechanism leads to changes in the physical and chemical properties of the soil particle surface, and the adsorption force for water molecules decreases. In the macroscopic aspect, it is specifically manifested as an increase in the free water content between soil particles, and the fluidity of solidified soil is enhanced. However, excessive modifiers formed an overly thick adsorption layer on the surface of soil particles, causing the solidified soil to undergo flocculation reactions. As a result, the fluidity of the fluid-solidified soil decreased instead.

(2) Curing agent

The relationship curve of fluid-solidified soil fluidity varies with the content of the curing agent, which is shown in Figure 8b. With the increase in the contents of mineral powder and fly ash, the fluidity of the fluid-solidified soil changes little within the range of 210–220 mm. It is worth noting that when the content of the curing agent is 10‰, the fluid-solidified soil fluidity reaches a maximum of 220 mm. These results collectively indicate that the variation in the dosage of the curing agent has a relatively small impact on the fluidity of the fluid-solidified soil. This is because the proportion of the curing agent in the solidified soil is relatively small, so the water requirement is relatively low compared with soil particles.

#### 3.3.2. The Impact Factors of Fluid Solidified Soil’s Compressive Strength

**Figure 9:**
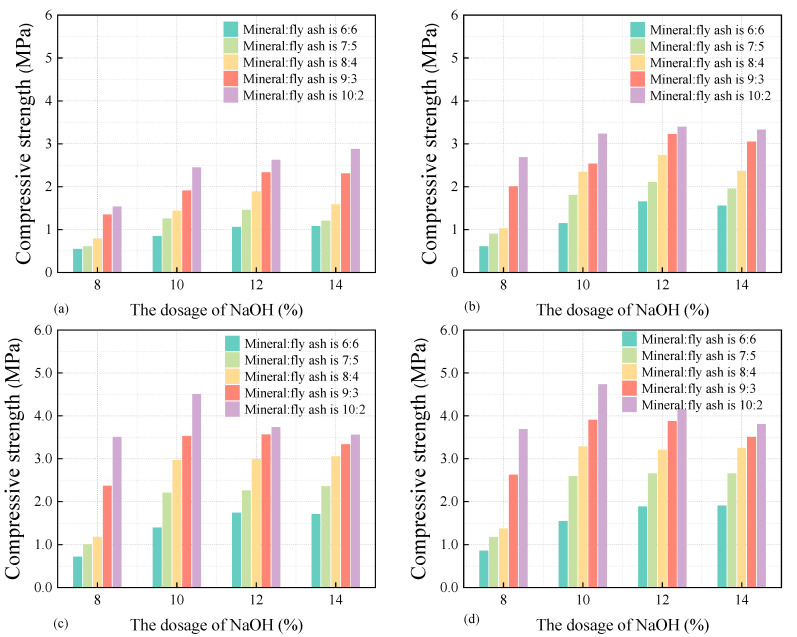
The curve of solidified soil compressive strength vs. additives dosage: (**a**) 3 d; (**b**) 7 d; (**c**) 14 d; (**d**) 28 d.

Figure 9 shows the curve of the compressive strength of fluid-solidified soil at different ages varies with the content of slag particles and sodium hydroxide. The compressive strength of the fluid-solidified soil gradually increases with the increase in curing time. The compressive strength at 7 d of curing can reach more than 65% of the strength of 28 d, while the compressive strength of the solidified soil after 14 d of curing can exceed 90% of the strength of 28 d. The analysis of the fluid-solidified soil prepared when the particle ratio of slag to fly ash is 9:3 and the dosage of sodium hydroxide is 10% shows that the compressive strengths after curing for 7 d, 14 d, and 28 d are 2.53 MPa, 3.52 MPa, and 3.9 MPa, respectively. That is, the strength of the solidified soil after 7 d and 14 d of curing can reach 75% and 90.2% of the 28 d strength, respectively, which indicates that the fluid-solidified soil has a tendency for early strength development.

(1) Granulated blast furnace slag dosage

The compressive strength of fluid-solidified soil shows a proportional upward trend with the increase in mineral powder content. Under the constant sodium hydroxide dosage of 10%, when the ratio of mineral powder to fly ash changed from 6:6 to 10:2, the compressive strength of the fluid-solidified soil after 28 days of curing increased from 1.54 MPa to 4.73 MPa, significantly improving by approximately 207%.

From a chemical perspective, the mineral powder is composed of a large amount of CaO, Al_2_O_3,_ and SiO_2_, among which CaO is the main component. Fly ash is mainly composed of Al_2_O_3_ and SiO_2_, among which SiO_2_ is the main component. Under the same application conditions, the chemical bonds in calcium oxide tetrahedra are more likely to be broken than those in silicon oxide tetrahedra. Furthermore, under the excitation effect of sodium hydroxide, the Ca^2+^ contained in the mineral powder can react with [SiO(OH)_3_]^−^ and [Al(OH)_4_]^−^ in the solidified soil to generate a large amount of C-S-H, C-A-H, and C-A-S-H gels, thereby significantly improving the compressive strength of the material. Through in-depth research on the influence law of the ratio of mineral powder to fly ash on the compressive strength of solidified soil, it can be known that the optimal ratio of mineral powder to fly ash in fluid solidified soil is 10:2.

(2) Sodium hydroxide dosage

The results show that when the ratio of mineral powder to fly ash remains unchanged at 10:2, with the increase in sodium hydroxide content, the compressive strength of fluid-solidified soil shows a trend of increasing first and then decreasing. Among them, when the content of sodium hydroxide is 10%, the compressive strength of the fluid-solidified soil is the greatest, which is 4.73 MPa. Afterwards, with the increase in the slag dosage, its compressive strength gradually decreases. This indicates that the compressive properties of fluid-solidified soil are relatively sensitive to the dosage of sodium hydroxide.

From the observations above, it can be inferred that the increase in sodium hydroxide dosage aggravates the polymerization reaction, engendering a greater output of gel products that effectively enhance fluid-solidified soil’s compressive strength. Nonetheless, an excess of sodium hydroxide may impede polymerization progression, as documented in studies [27,28,29], undermining the formation of C-S-H gel [30]. Moreover, surplus Na^+^ in the mud may unite with active silicon and active aluminum, engendering a larger quantity of low-strength N-A-S-H gel. This, in turn, weakens the bond between active silicon, active aluminum, and Ca^2+^, resulting in a diminished presence of high-strength calcium silicate and calcium aluminates silicate gel, thus diminishing strength.

#### 3.3.3. The Impact Factors of Fluid Solidified Soil’s Self-Shrinkage

**Figure 10:**
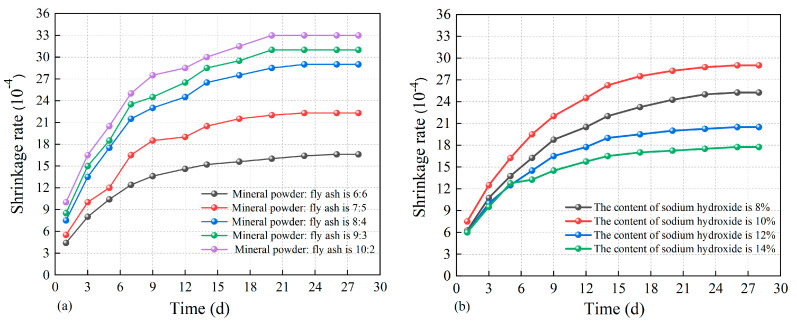
The curve of solidified soil self-shrinkage vs. additives dosage: (**a**) granulated blast furnace slag; (**b**) sodium hydroxide.

Self-shrinkage refers to the macroscopic volume reduction in the soil after solidification, which can be expressed by the self-shrinkage rate. Figure 10 shows the curve of the self-shrinkage of fluid-solidified soil varies with the content of admixtures.

(1) Granulated blast furnace slag dosage

It can be seen from Figure 10a that the self-shrinkage rate of fluid-solidified soil increases with the increase in mineral powder content. With the ratio of slag to fly ash increasing from 6:6 to 7:5 and 8:4, the maximum self-shrinkage rate of fluid-solidified soil increased from 16.5 × 10^−4^ to 22.3 × 10^−4^ and 29.0 × 10^−4^, increasing by 35.2% and 75.7%, respectively. When the ratio of slag to fly ash was adjusted to 9:3 and 10:2, the maximum self-shrinkage rates were 31.0 × 10^−4^ and 33.0 × 10^−4^, respectively. It is worth noting that when the ratio of granulated slag to fly ash exceeds 9:3, the growth rate of the self-shrinkage rate of solidified soil slows down significantly.

The reason for this phenomenon is that the mineral powder particles undergo a chemical reaction under the excitation of alkali to form hydrated aluminate silica gel. With the increase in mineral powder content, the intensity of alkali-activated polymerization increases, resulting in a sharp increase in hydration products, thereby enhancing the self-shrinkage rate of solidified soil.

In addition, the filling effect of fly ash is noteworthy. Due to its limited reactivity, fly ash is difficult to activate rapidly when the slag is abundant. Therefore, in the initial stage of the reaction, fly ash acts as an inert sand-like substance. It occupies the interstices amid the cementitious materials and soil particles, thereby functioning as a physical dispersant within the composite material system. This effect alleviates the self-shrinking tendency of hydration products to a certain extent.

(2) Sodium hydroxide content

It can be seen from Figure 10b that the self-shrinkage rate of the fluid-solidified soil shows a changing trend of increasing first and then stabilizing over time. However, under the same time conditions, the self-shrinkage rate of solidified soil is closely related to the dosage of sodium hydroxide. An increase in sodium hydroxide content from 8% to 10% leads to a growth in the maximum self-shrinkage rate of fluid-solidified soil from 25.5 × 10^−4^ to 29.0 × 10^−4^, signifying a 13.7% augmentation. However, as the sodium hydroxide content escalates from 10% to 12% and 14%, the maximum self-shrinkage rate experiences a reduction from 29.0 × 10^−4^ to 20.5 × 10^−4^ and 17.7 × 10^−4^, translating to decreases of 29.3% and 38.9%, respectively. These test outcomes demonstrate that the self-shrinkage behavior of fluid-solidified soil exhibits an initial increase followed by a subsequent decrease with escalating sodium hydroxide content.

The self-shrinkage rate of solidified soil shows the characteristic of increasing first and then decreasing with the increase in sodium hydroxide dosage, which can be attributed to the following reasons. The increase in sodium hydroxide content can enhance the solubility of active silica and alumina in solidified soil, thereby raising their self-shrinkage rate. However, excessive sodium hydroxide can inhibit the formation of C-S-H and C-A-S-H gels. Meanwhile, excessive Na^+^ promotes the overproduction of N-A-S-H gel, thereby causing changes in the pore structure of the solidified soil. Therefore, the development trend of self-shrinkage of fluid-solidified soil has reversed. When the sodium hydroxide content is 10%, the self-shrinkage rate of the solidified soil reaches its maximum value.

## 4. Conclusions

In light of the challenges associated with utilizing waste mud containing high moisture content, this study focuses on investigating the impact of deep dehydration on waste mud and the long-term properties of geopolymer fluid-solidified soil. The results show that the waste mud can be used to prepare fluid-solidified soil after deep dewatering, providing valuable insights for further achieving the recycling of resources. The relevant conclusions are as follows:

(1) In comparison to inorganic flocculants, organic flocculants exhibit an even more pronounced effect on the separation of mud and water. This can be attributed to the enhanced adsorption properties of the flocculant and polymer chains when stretched in water, resulting in increased gravitational potential energy and accelerated separation between mud and water. The optimal dosage of APAM flocculation for treating waste mud was determined to be 0.25 g/L, which can effectively reduce the water content to 90%.
(2) The deep dewatering of waste mud can be achieved through independently innovated vacuum filtration combined with an electroosmosis test device, among which the combination of independent anode vacuum filtration and electroosmosis dehydration is the most effective. This is because under the effect of electroosmosis, soil particles, and water molecules accelerate their movement, which increases the dissipation of pore water pressure in the center and bottom pore spaces of the flocculated mud. By combining electroosmosis with vacuum filtration, a greater pore pressure dissipation can be achieved in a single vacuum-filtered flocculent mud, particularly during later stages of consolidation when it comes to dissipating pore water pressure in the relatively dense subsoil.
(3) The properties of geopolymer fluid-solidified soil prepared from dehydrated mud are significantly influenced by its composition. With an increase in granulated blast furnace slag and sodium hydroxide, the compressive strength and shrinkage of the fluid-solidified soil are enhanced. The optimal mix ratio of solidified soil is 10:2 for mineral powder to fly ash, and the dosage of sodium hydroxide is 10%.
(4) The deeply dewatered waste mud can be effectively utilized in the production of flowable backfill materials, thereby promoting resource recycling and providing valuable insights for the further reuse of waste mud. According to actual engineering calculations, when preparing fluid-solidified soil with conventional cement, the proportion of cement cost is the highest (about 30–60%), with a total cost of approximately RMB 38–227 yuan/m^3^. However, using industrial dewatering mud instead of cement can significantly reduce costs. Under the same conditions, the total cost is approximately RMB 18–122 yuan/m^3^, which is 55.95% lower than the former. It can significantly improve economic benefits. In addition, the use of fluid-solidified soil helps reduce carbon emissions and energy consumption, achieving sustainable and zero-pollution utilization of industrial waste mud.

## Figures and Tables

**Table 1:** The test scheme of vacuum filtration combined electroosmosis dehydration.

Test	Test Conditions	Voltage	Vacuum Degree
ZD1	Vacuum filtration dewatering and consolidation	0 V	−80~−90 kPa
ZD2	Vacuum filtration combined with independent anode synchronous consolidation	30 V	−80~−90 kPa
ZD3	Vacuum filtration combined with composite anode synchronous consolidation	30 V	−80~−90 kPa

**Table 2:** Test scheme for influencing factors of fluid-solidified soil fluidity.

Testing Index	Number	Curing Agent (‰)	Modifier (‰)
Fluidity	1-I-1	6.0	0.0
	1-I-2		1.0
	1-I-3		2.0
	1-I-4		3.0
	1-I-5		4.0
	1-I-6		5.0
	1-II-1	6.0	3.0
	1-II-2	8.0	
	1-II-3	10.0	
	1-II-4	12.0	
	1-II-5	14.0	
	1-II-6	16.0	
	1-II-7	18.0	

**Table 3:** Test scheme for influencing factors of fluid-solidified soil compressive strength and self-shrinking.

Testing Index	Number	Sodium Hydroxide (%)	Furnace Slag:Fly Ash
Compressive strength and Self-shrinking	2-I-1	8	6.0:6.0
	2-I-2		7.0:5.0
	2-I-3		8.0:4.0
	2-I-4		9.0:3.0
	2-I-5		10.0:2.0
	2-II-1	8	6.0:6.0
	2-II-2	10	
	2-II-3	12	
	2-II-4	14	

## Data Availability

The original contributions presented in the study are included in the article, further inquiries can be directed to the corresponding author.
